# Prenatal detection of right aortic arch

**DOI:** 10.1007/s00404-019-05056-5

**Published:** 2019-01-31

**Authors:** Gülen Yerlikaya, Tünay Efetürk, Stephanie Springer, Theresa Reischer

**Affiliations:** 1 Department of Gynecology and Obstetrics, Division of Feto-Maternal Medicine, Medical University of Vienna, Waehringer Guertel 18-20, 1090 Vienna, Austria; 2 Department of Obstetrics and Gynaecology, The Royal London Hospital, Barts Health NHS Trust, Women’s Health, London, UK

**Keywords:** Right aortic arch, 22q11.2 microdeletion, Fetal MRI, Prenatal

## Abstract

**Purpose:**

To examine an unselective population of fetuses with right aortic arch (RAA) and suggest perinatal management. Second, to evaluate the importance and possible implication of fetal MRI in those cases.

**Methods:**

Retrospective study of 36 patients with RAA diagnosed prenatally between 2006 and 2017 in a tertiary referral center.

**Results:**

32 fetuses were diagnosed with RAA and 4 with double aortic arch (DAA). 7 (19, 5%) cases had intracardiac abnormalities. Tetralogy of the Fallot was the most frequent one. Other extracardiac malformations were observed in 11/36 (30, 6%). Karyotype was available in 16 (44, 5%) cases. Two had 22q11.2 microdeletion, two trisomy 21, and one 20p12.2 duplication. Two needed surgery for respiratory symptoms. A newborn was identified with epilepsy, Lennox–Gastaud syndrome and Pallister–Killian syndrome postnatally and another one with showed hyperreflexia and premature closer of the fontanelle. Three feticides were performed for pregnancy termination in one case with 22q11 deletion, trisomy 21, and partial agenesis of corpus callosum.

**Conclusion:**

RAA can be detected by fetal echocardiography and it is associated with other cardiac or non-cardiac abnormalities, 22q11 microdeletion, trisomy 21, and other chromosomal abnormalities. karyotyping should be offered in any case of RAA, irrespective of co-existing malformations. Fetal MRI is a promising diagnostic tool for assessment of congenital cardiovascular abnormalities and extracardiac anomalies.

## Introduction

In right aortic arch the transverse arch is to the right of the trachea, in contrast to the normal left aortic arch. The incidence of right aortic arch (RAA) has been reported as 1 in 1000 in low risk-population, although the exact incidence is unknown [1, 2]. There can be various forms of aortic arch abnormalities, such as right-sided aortic arch with right ductus arteriosus and with mirror image branching, RAA with aberrant left subclavian or innominate artery; right aortic arch with left ductus arteriosus (DA) and double aortic arch (DAA).

The diagnosis of right-sided aortic arch is important due to possible associated cardiac and non-cardiac defects and chromosomal abnormalities, especially 22q11 microdeletion and trisomy 21 [3]. RAA is best detected in the three-vessel-trachea view.

The aim of this study was to review fetuses with the diagnosis of RAA in our center to evaluate associated conditions and outcome and, therefore, to suggest perinatal and postnatal management. Second, evaluate the importance and possible implication of fetal MRI in those cases.

## Methods

**Fig. 1:**
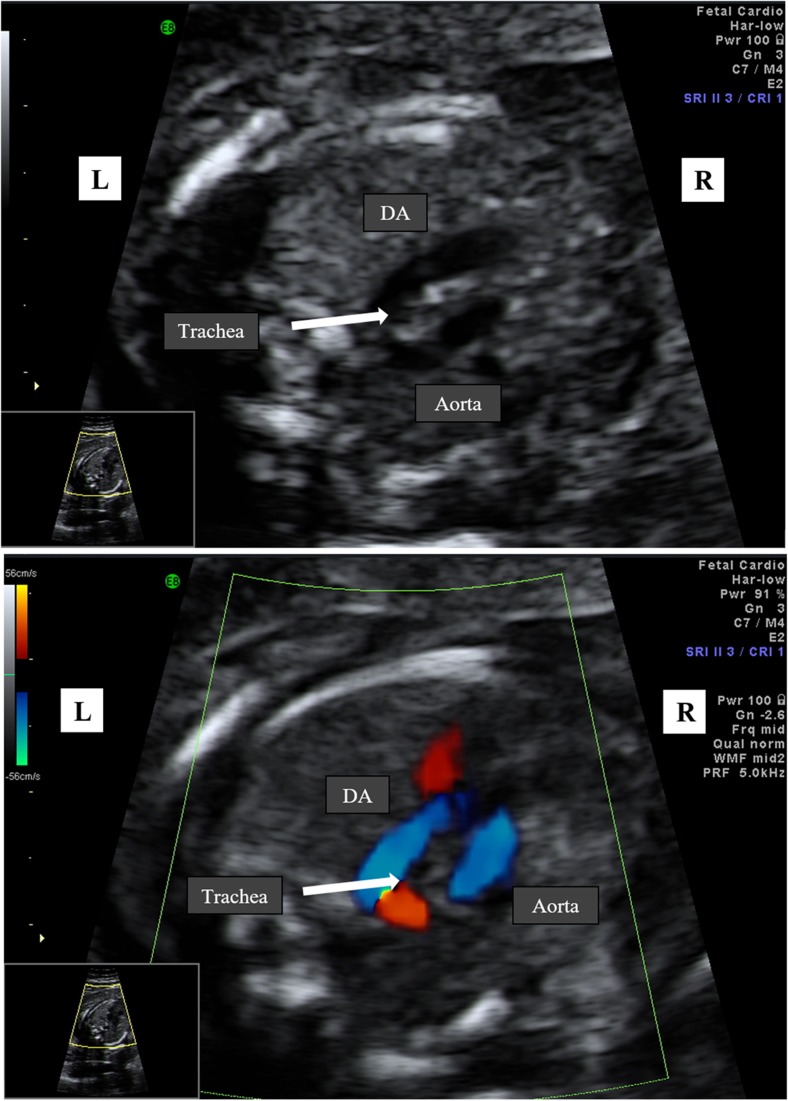
**a** Right aortic arch at 21 weeks of gestation in gray scale mode. The transverse arch is on the right of the trachea with a left ductus arteriosus (DA). The aortic arch and DA form a U-shaped configuration with the trachea between the two vessels (U-sign). **b** Right aortic arch with color Doppler at 21 weeks of gestation

In this retrospective study, all cases between 2006 and 2017 were enrolled (Table 1). We searched our database for all cases of a RAA. Ultrasound examinations were performed on a Voluson E6 and E10 (GE Medical Systems, Zipf, Austria). A right-sided aortic arch was diagnosed when the transverse arch was to the right of the trachea in the three-vessel-trachea view. Subsequently, the aortic arch and DA form a U-shaped configuration with the trachea between the two vessels (so-called U-sign) (Fig. 1a, b). In almost all the cases, the DA is left sided. In cases of a right-sided DA, the aortic arch and the duct are placed to the right of the trachea and form a V-shaped configuration. All the cases of RAA were examined by a subspecialist in fetal or fetal–maternal medicine to confirm the diagnosis and whether there were any other malformations present. The fetal thymus was not examined in cross-sectional planes. Fetal MRI was performed for additional information concerning possible intra- and extracardiac abnormalities. Perinatal and postnatal echocardiographic examination was performed through a specialist in fetal echocardiography. Karyotyping for diagnosis of 22q11 deletion was offered to each patient. The outcomes observed were associated intracardiac and/or extracardiac malformations and neonatal outcome related to associated malformations, chromosomal abnormalities, such as 22q11 microdeletion and trisomy 21. Postnatal symptoms related to compression of trachea/esophagus and surgery due to vascular ring symptoms. Additional data, such as gestational age at diagnosis and fetal and neonatal outcome were also collected.

**Table 1:** Summary of 36 cases with right aortic arch

Case	GA at diagnosis (week)	Aortic arch	DA	Intracardiac anomalies	Extracardiac anomalies	Karyotype	Outcome	Surgery	Postnatal findings
1	20	RAA	L	TOF	No	46, XY	Livebirth	For TOF	No
2	28	RAA	L	No	Mild ventriculomegaly, shortening of the long bones	n.k	Livebirth	No	Epilepsy, Lennox–Gastaud syndrome, Pallister–Kilian syndrome
3	23	RAA	L	No	SUA	n.k	Livebirth	No	No
4	18	RAA	L	No	No	46, XX	Livebirth	No	No
5	23	RAA	L	No	No	n.k	Livebirth	No	Hyperreflexia, premature closer of the fontanelle, neurological development normal until now
6	24	RAA	L	No	Hypoplastic NB	Trisomy 21	TOP		
7	24	RAA	L	No	No	46, XX	Livebirth	Yes for VSD	Perimembraneous VSD with mitral regurgitation and pulmonary hypertension due to right–left shunt
8	21	RAA	L	No	No	n.k.	Livebirth	No	Trisomy 21
9	20	DAA	L	No	No	46, XX	Livebirth	No	VSD
10	23	DAA	L	No	No	46, XX	Livebirth	No	No
11	22	RAA	L	No	Talipes bilateral	22q11.2	TOP		
12	22	RAA	L	No	No	n.k.	Livebirth	No	22q11.2
13	22	RAA	L	No	No	n.k.	Livebirth	No	No
14	20	RAA	L	No	No	46, XX	Livebirth	No	No
15	22	RAA	L	Persistent left superior vena cava	SUA, caudal regression syndrome with tethered cord, hydronephrosis	n.k	Livebirth	Yes due to multiple malformtaion	VACTERL association
16	21	DAA	L	TGA	No	n.k	Livebirth	Yes	RAA, ASD II, anomalous pulmonary connection, aplasia of the thumb, ear and clavicula, chylothorax, karyotyping normal
17	25	RAA	L	No	No	n.k	Livebirth	No	No
18	24	RAA	L	No	No	n.k	Livebirth	No	No
19	24	RAA	L	VSD	Partial agenesis of corpus callosum Diffus cortical development disorder of the brain	n.k	TOP		
20	22	RAA	L	No	No	Tr21			Still pregnant
21	21	RAA	L	No	No	n.k.	Livebirth	No	No
22	23	RAA	L	No	No	Mosaic 21	Livebirth	No	46, XX
23	21	RAA	L	No	No	46, XX	Livebirth	No	No
24	15	RAA	L	No	SUA	20p12.2 duplication	Livebirth	No	No
25	21	RAA	L	No	No	46, XX	Livebirth	No	No
26	26	RAA	L	No	No	n.k.	Livebirth	Yes	No
27	21	RAA	L	No	No	46, XY	Livebirth	No	No
28	23	RAA	L	No	No	n.k.	Livebirth	No	No
29	21	RAA	L	No	No	n.k.	Livebirth	No	No
30	29	DAA	L	No	No	n.k.	Livebirth	?	No
31	21	RAA	L	No	No	n.k.	Livebirth	No	No
32	20	DAA	L	No	No	46, XY	Livebirth	No	No
33	20	RAA	L	No	No	n.k	Livebirth	Yes	DAA
34	23	RAA	L	TOF	SUA, mild retrognathia	n.k	Livebirth	Yes for TOF	No
35	23	RAA	L	No	Hypoplasia of pontocerebellum	n.k	Livebirth	No	No
36	27	RAA	L	No	No	46, XX	Livebirth	No	No

*GA* gestational age, *DA* ductus arteriosus, *RRA* right aortic arch, *DAA* double aortic arch, *L* left, *TOF* tetralogy of fallot, *SUA* single umbilical artery, *n.k.* not known, *VSD* ventricular septal defect, *TGA* transposition of great arteries, *TOP* termination of pregnancy

The study obtained ethics approval from the Medical University’s Ethics Committee of Vienna (EK Nr: 2062/2017). Due to the observational and retrospective nature of the study consent for participation by parents was not required.

## Results

During this period, in total 36 fetuses had aortic arch abnormalities. 32 fetuses were diagnosed with RAA and 4 with DAA. The mean gestational age was 22 (range 15–29). The mean maternal age at the time point of diagnosis was 27 (range 20–38; for detailed characteristic description of the study cohort see Table 2). 33 cases were in singleton pregnancies and 3 in dichorionic–diamniotic twin pregnancies. All fetuses had a left-sided duct. No right-sided duct was described in our study population. Three out of four DAA were confirmed postnatally. In one of the 32 suspected right-sided aortic arch, the diagnosis turned out to be a DAA. Seven (19. 5%) cases had intracardiac abnormalities. Five were detected prenatally (two tetralogy of fallot one with pulmonary atresia, TGA, persisted left superior vena cava and one VSD) and two after birth (Table 1). Prenatally one DAA presented transposition of the great arteries, which could not be confirmed after birth. Instead it was described as a RAA with atrioventricular septal defect and total anomalous pulmonary connection and in one case a perimembraneous VSD with mitral regurgitation and pulmonary hypertension due to right–left shunt was diagnosed, which needed operation. Another simple VSD was seen postnatally but no intervention was necessary yet. Fetal MRI gave additional information about extracardiac malformation, such as hypoplasia of the cerebellum in one fetus and caudal regression syndrome with tethered cord and VACTERL association in another fetus. Both were confirmed after birth. Other extracardiac malformations were observed in 11/36(30. 6%) of patients. Nine prenatally, whereas two were diagnosed postnatally. The most common abnormality prenatally was single umbilical artery (SUA) in four fetuses. Ventriculomegaly and shortening of the long bones in one, severe hydronephrosis, retrognathia in one fetus, partial agenesis of corpus callosum and cortical development disorder in one, talipes in one fetus and one absent nasal bone (Table 1).

**Table 2:** General characteristics of the study cohort

Characteristics	Value
Maternal age, years	27 (range 20–38)
Gestational age at diagnosis, weeks	22 (range 15–29)
Extracardiac anomalies	11/36 (30.6%)
Intracardiac anomalies	7/36 (22.2%)
Chromosomal anomalies	5/36 (13.9%)
22q11.2deletion	2/36 (5.6%)
Trisomy 21	3/36 (8.3%)
Other (+Syndromes)	2/36 (5.6%)
Termination of pregnancy	3/36 (8.3%)
Surgery due to vascular ring symptoms	2/36 (5.6%)

Karyotype was available in 16(44.4%) out of 36 cases. Karyotyping was declined in the other cases. In the group where an invasive procedure was performed 22.q11 deletion was present in 1 case. Furthermore, trisomy 21, 20p12.2 duplication and one mosaic for trisomy 20. Postnatally the genetic testing for one fetus with mosaic for trisomy 20 showed normal karyotype. In the other group, without karyotyping, one case was diagnosed with 22q11 deletion and one with trisomy 21 in neonatal period due to phenotypic appearance. A third case was identified with epilepsy, Lennox–Gastaud syndrome and Pallister–Killian syndrome postnatally. And another case with isolated RAA showed hyperreflexia and premature closer of the fontanelle. The reason is unknown till yet but neurological development is normal so far. Postnatal examination of all other newborns did not show any phenotypic abnormalities and, therefore, they were considered to be normal, without any chromosomal abnormalities or genetic syndromes. One woman is still pregnant, therefore, the final outcome is not known at this time point.

Two needed surgery after they were borne due to airway obstruction. In both cases it was described as RAA with left-sided DA and an aberrant left subclavian artery, arising from a remnant of the primordial aortic arch, also known as Kommerell‘s diverticulum. Three feticides were performed for pregnancy termination in one case with 22q11 deletion, trisomy 21, and partial agenesis of corpus callosum.

## Discussion

RAA can be detected prenatally in the three-vessel-trachea view, but the exact incidence of this condition still remains unknown. We did not have any cases of a right-sided aortic arch with a right-sided duct. It is assumed that some cases of RAA with right-sided DA may have been missed and as a consequence the incidence of complete right-sided variants and RAA in general may be underreported in our population. However, we still could show clearly that RAA is associated with additional intra and extracardiac abnormalities. In comparison to other reports we performed fetal MRI in each case for additional information. To the best of our knowledge there are almost no report about RAA and fetal MRI in the literature. Fetal MRI did not just confirm the diagnosis which has been made by ultrasound it detected also malformations which were missed beforehand such as pontocerebellar hypoplasia in one fetus and multiple abnormalities in another case with caudal regression syndrome, severe hydronephrosis, micropenis, SUA, and persistent superior left vena cava, hypotelorismus, and suspicion of VACTERL syndrome. We believe that fetal MRI is a useful adjunct for assessing the fetal anatomy, especially in cases of isolated aortic arch abnormalities. Li et al. could show that fetal cardiovascular magnetic resonance (CMR) imaging, particularly with transverse views at the level of the aortic arch, in the diagnosis of aortic arch anomalies had an accuracy of 95.6% [4]. Fetal CMR is a promising diagnostic tool for assessment of congenital cardiovascular abnormalities, especially in situations that limit echocardiography [5]. On the contrary, it is believed that the suspicion of a right aortic arch in an asymptomatic baby does not justify magnetic resonance imaging, although it is the best modality for demonstrating the arch vessels [6].

Right aortic arch is associated significantly often with 22q11.2 deletion and trisomy 21 as reported previously [3, 7]. We had two cases with 22.q11.2 deletion, and two with trisomy 21, whereas two of these conditions were diagnosed after birth. Furthermore, we had one newborn diagnosed with epilepsy, Lennox–Gastaud syndrome and Pallister–Killian syndrome and another case with hyperreflexia and premature closer of the fontanelle. All this cases happened in the group of women who declined invasive procedure. It is unclear in professional circles whether karyotyping should be performed with array or FISH or not for isolated findings of RAA. It has been reported that in cases with isolated RAA or DAA up to 24% have 22q11.2 microdeletion postnatally [8]. The overall percentage of chromosomal abnormalities in our study was 13.9% with 22q11 microdeletion being responsible for roughly half the cases (5.6%). However, the severity of trisomy 21 and especially 22q11.2 deletion is unpredictable and, therefore, even in the absence of other malformation the prognosis is uncertain. Especially the severity of immunodeficiency, development impairment, and mental and psychiatric illness are unpredictable. As a consequence many parents will probably request further testing. These facts have to be made clear for the couple and second, according to our findings, we could show that isolated RAA can be associated with other chromosomal abnormalities. It seems reasonable to mention the importance of further interventions in isolated right-sided aortic arch. However, some authors have suggested that RAA as an isolated finding in a fetus may not justify karyotyping [9] and other authors believe that the identification of the thymus hypoplasia or aplasia may help to identify the fetuses at higher risk for 22q11.2 microdeletion [10]. We did not evaluate the thymus in our retrospective study. This and that karyotyping was not performed for each case may be one limitation in our study which may have underestimated the incidence of 22q11.2 microdeletion cases. However, it is worth mentioning that 22q11.2 was not the only chromosomal anomaly we found in our population, as it was the case in other studies [11]. It was associated with trisomy 21 as well and 20p12.2 duplication. Examination after birth by a skilled neonatologist was uneventful and, therefore, we concluded that 20p12.2 duplication in this case has unknown clinical significance.

Another limitation of our study was the fact that it was retrospective, although postnatal confirmation and follow-up and fetal MRI for each case were available. However, we still could show that aortic arch abnormalities such as RAA can be associated with different chromosomal and non-chromosomal abnormalities and syndromes apart from 22q11.2 deletion and trisomy 21, which are well known. Nevertheless, a larger number of cases is necessary to provide more accurate information on the incidence of RAA and associated malformations.

## Conclusion

RAA is associated with other cardiac and non-cardiac malformation, microdeletion 22q11, and trisomy 21. Fetal MRI is a good tool for diagnosis of aortic arch anomalies and intra and extracardiac malformations. Furthermore, detailed fetal cardiac and extracardiac examination and fetal echocardiography prenatally and postnatally should be undertaken in all cases of RAA. Based on our results we would recommend that karyotyping should be offered in any case of RAA, irrespective of co-existing malformations. This information we received through the retrospective study may be considered to prepare a possibly standardized approach to RAA. However, a much larger numbers of cases are needed to do so.
